# Community-Based ART Programs: Sustaining Adherence and Follow-up

**DOI:** 10.1007/s11904-016-0335-7

**Published:** 2016-10-13

**Authors:** Joia S. Mukherjee, Danika Barry, Robert D. Weatherford, Ishaan K. Desai, Paul E. Farmer

**Affiliations:** 1 Division of Global Health Equity, Brigham and Women’s Hospital, 641 Huntington Avenue, Boston, MA 02115 USA; 2 Partners In Health, 888 Commonwealth Avenue, Boston, MA 02215 USA; 3 Department of Global Health and Social Medicine, Harvard Medical School, 641 Huntington Ave, Boston, MA 02115 USA

**Keywords:** Antiretroviral therapy, Community-based, Decentralization, Adherence, Community health workers, Health systems strengthening, Accompaniment, Partners In Health, HIV care, Retention

## Abstract

The advent of antiretroviral therapy (ART) in 1996 brought with it an urgent need to develop models of health care delivery that could enable its effective and equitable delivery, especially to patients living in poverty. Community-based care, which stretches from patient homes and communities—where chronic infectious diseases are often best managed—to modern health centers and hospitals, offers such a model, providing access to proximate HIV care and minimizing structural barriers to retention. We first review the recent literature on community-based ART programs in low- and low-to-middle-income country settings and document two key principles that guide effective programs: decentralization of ART services and long-term retention of patients in care. We then discuss the evolution of the community-based programs of Partners In Health (PIH), a nongovernmental organization committed to providing a preferential option for the poor in health care, in Haiti and several countries in sub-Saharan Africa, Latin America, Russia and Kazakhstan. As one of the first organizations to treat patients with HIV in low-income settings and a pioneer of the community-based approach to ART delivery, PIH has achieved both decentralization and excellent retention through the application of an accompaniment model that engages community health workers in the delivery of medicines, the provision of social support and education, and the linkage between communities and clinics. We conclude by showing how PIH has leveraged its HIV care delivery platforms to simultaneously strengthen health systems and address the broader burden of disease in the places in which it works.

## Introduction

Over the last twelve years, since the establishment of the Global Fund to Fight AIDS, Tuberculosis and Malaria (2002) and the President’s Emergency Plan for AIDS Relief (2003), access to HIV treatment has been expanded to millions of people across the globe [1, 2]. Adherence to the daily schedule of antiretroviral therapy (ART) and lifelong retention in care are both critical to building strong HIV prevention and treatment programs and achieving optimal patient outcomes. Yet, adherence to any therapy is determined by a complex matrix of factors affecting a patient’s ability to take medications and a health system’s ability to facilitate access to high-quality care [3]. To address patients’ social and economic needs and overcome structural barriers to good health, community-based, decentralized care became the norm for HIV care delivery in the global scale-up of ART. The success of community-based, decentralized care had by 2015, resulted in the enrollment of over 15 million people on ART [4] and has spawned efforts to leverage disease-specific health initiatives to comprehensively strengthen health systems. This paper reviews the recent literature on community-based HIV care in low- and low-to-middle-income country settings and describes the community-based programs of the nongovernmental organization Partners In Health (PIH).

## Community-Based ART: a Review of the Recent Literature

In the last 2 years, several articles examining the efficacy of community-based ART have been published. The data for this review were identified by searching PubMed, MEDLINE, and reference lists of eligible studies in any language published between January 1, 2014 and January 12, 2016. Studies included in this review were those published in the last 10 years that described or evaluated community based ART ptrogram in low or low-middle incomce countries. Evidence generated form PIH-supported programs will be discussed following this reivew. In addition, as a pioneer of the community-based approach to ART [5–9], PIH, along with its public sector partners, has used its platforms for the delivery of HIV care to address the broader disease burden in the communities in which it works.

## Principles of Community-Based Therapy

Community-based approaches to ART, while varied and uniquely adapted across diverse settings, share two key principles: decentralization of care and retention in care. Decentralization involves moving care closer to the patient by offering ART and related health and social services at primary care clinics, through community distribution posts, or in patient homes. Efforts to decentralize HIV care have required using simplified regimens and algorithmic clinical approaches and capacitating a broad set of workers to deliver ART. The second principle, community participation in the promotion of retention and adherence to medication, is focused on home-based follow-up and psychosocial and peer support. There are a variety of approaches to improving adherence to ART, including the use of community health workers (CHWs), peer counselors, and support groups; the provision of food and nutritional support; the coverage of transportation fees and school fees for children; and forms of psychosocial support. With many countries approaching over a decade of experience scaling up ART and achieving good, clinical outcomes, these two principles—the decentralization of care and the establishment of programs to foster retention and adherence—have been underscored in the recent literature on community-based ART.

### Decentralization of Care

During the past 13 years, the decentralization of ART beyond capital cities and into primary care clinics has achieved remarkable results. A 2014 study of the decentralization of HIV care in Ethiopia notes that by 2013, over 900 health facilities were providing ART across the country, increasing the number of patients who had ever been initiated on ART to 439,000 (from just 9000 in 2005) and reaching over 300,000 patients currently on treatment (about 75 % ART coverage). The investigators attribute Ethiopia’s successful decentralization to several factors, including a public health approach, which they define as standardizing treatment regimens, training diverse cadres of health workers, and simplifying monitoring systems; the expansion of HIV testing and counseling; community engagement; and the strengthening of health systems through, for example, investments in infrastructure, supply chain, laboratory capacity, human resources, and patient information systems [10].

Reaching rural populations and those facing structural impediments to health care—importantly the financial and time costs of clinic visits—has also been a widely cited goal of community-based ART programs. Abongomera and colleagues analyzed health service usage in poor, rural parts of northern Uganda and showed through surveys and interviews that HIV-infected people travel considerable distances on foot to access HIV services but seek treatment for common conditions locally; they hypothesize that decentralizing ART coverage to rural primary care facilities would substantially improve access and mitigate poor patient outcomes [11]. To expand the reach of ART, care has often been moved beyond the clinic walls to community distribution points and mobile posts [12] and even into homes. For example, Médecins Sans Frontières (MSF) has worked with several national health ministries in sub-Saharan Africa to develop novel strategies to expand and optimize ART delivery: reducing the frequency of clinic visits by using CHWs to distribute ART refills, providing ART and consultations at patient health clubs, establishing ART distribution points run by people living with HIV, and organizing patient-led community ART groups. An evaluation of MSF’s strategies concluded that they “lightened the burden for both patients (reduced travel and lost income) and health system (reduced clinic attendance)” [13]. Similar studies from Haiti, Mozambique, Tanzania, Zambia, and Uganda support the efficacy of community-supported distribution [14–16]. Linking HIV diagnosis (including the use of rapid diagnostics) with home- or community-based ART initiation offers further opportunity for increased coverage: a randomized trial of 16,660 Malawian adults documented a significant increase in the rate of ART initiation among those who were offered optional home initiation of HIV care after HIV self-testing (OraQuick ADVANCE® Rapid HIV-1/2 antibody test, OraSure Technologies) as compared with those offered only facility-based services after self-testing [17]. A recent meta-analysis published in *Nature* showed that community-, mobile-, and home-based testing reached a higher number of first-time testers, men, and young adults than did clinic-based testing; if connected to HIV treatment services, the study concludes, community-based testing can increase ART initiation and coverage [18].

Task shifting is another cornerstone of decentralization. In 2014, a Cochrane review was published to compare the outcomes of physician-led ART delivery with those of ART delivery involving other cadres of health workers. Surveying the literature on task shifting for the initiation and maintenance of ART between 1996 and 2014, the review concluded—based on evidence from four randomized controlled trials and six cohort studies—that shifting the responsibility of HIV care from physicians to nurses or CHWs is safe, does not increase HIV-related mortality, and may even improve follow-up among those receiving care initiated by nurses [19]. A systematic review that used CD4 count or HIV viral load to compare different models of ART service delivery found decentralization and task shifting to be “effective strategies” and cited several articles showing that nurse-led ART and home- or community-based ART (delivered by trained community members) can yield outcomes similar to, or even superior to, those achieved by physician-led and facility-based delivery models, respectively [20].

### Retention in Care

In addition to spanning the geographic distance to clinics and hospitals and thus supporting decentralization, CHWs have helped retain HIV patients in care by extending the continuity of the care delivery pathway and offering long-term patient accompaniment. A study of CHWs in low-income, peri-urban communities of Cape Town, South Africa, found that CHWs conducted an average of six visits per day, each lasting an average of 9 min. Forty-six percent of their total work time was spent with patients (e.g., ensuring correct medication dosage, monitoring for side effects, providing social support), and the remainder was spent conducting “noncontact” activities such as walking and tracing patients who had defaulted from treatment [21]. Average walking time between patients in this peri-urban setting was about 8 min; in many rural settings, the geographic distance covered by CHWs is significantly larger.

Community-based ART programs can tackle some of the structural barriers that undermine retention in care. In Ethiopia, for example, while decentralization has supported initiation of ART [10], retention has varied significantly across health facilities. The highest retention rates have been achieved in health centers that adopt a package of retention-promoting activities such as “strong and coordinated defaulter tracing and outreach services” and the use of case managers and patient information systems to ensure continual, holistic patient care [22]. Community support groups that provide peer counseling to patients with HIV, and sometimes distribute ART have also been shown to promote adherence and long-term retention in care [23].

This is consistent with other findings that document the supportive effect of community programs on retention in care. Grimsrud and colleagues recently published two analyses of community-based adherence clubs in South Africa and showed their positive effects on both decentralization and retention in care (measured as loss to follow-up, which they defined as “having no visit in the first 12 weeks of 2014”); of 2133 patients with HIV who participated in community-based adherence clubs, only 6 % of participants were lost to follow-up after a period of 12 months [24, 25]. Similarly, a matched retrospective cohort study in 10 provinces of Mozambique demonstrated lower lost-to-follow-up rates among patients in Community Adherence and Support Groups than among those in individual care (11 versus 26 %) and lower overall attrition (12 versus 28 %), but similar mortality (1 % in both cohorts) [26]. Other studies have shown similar improvement in retention associated with community-based support but without clear mortality benefit [27, 28]. Only one recent study saw an improvement in mortality associated with community-based support, namely a descriptive case study of a community home-based care organization in Swaziland that provided individual and household support for ART adherence. In this study, cohort data from 993 care supporters with 3839 clients in 37 communities demonstrated a 71 % reduction in overall mortality (from 32 % in 2008 to 9 % in 2013), though the authors acknowledged their study was not designed to determine causality or statistical significance [29].

The benefits of community-based adherence programs have also been seen in pediatric populations. A large study of 4853 children on ART in Cape Town, South Africa, demonstrated that a “community-based adherence support” intervention had a marked effect on virologic suppression over standard of care without community-based support. About 20 % of the children received community-based adherence support, which included home visits by patient advocates to provide psychosocial support and address contextual household challenges affecting adherence. A significant difference in rates of virologic suppression was found: 65.6 % among those who received the intervention compared to just 55 % among those who did not [30].

## The Partners in Health Experience

PIH began community-based treatment of HIV with ART in 1998 in a rural clinic in central Haiti [5, 6]. In 2002, with the establishment of the Global Fund to Fight AIDS, Tuberculosis, and Malaria, PIH was afforded an opportunity to scale up its services and collaborate with Haiti’s public health authorities to adopt a community-based model to decentralize care from a single charity hospital to more than a dozen public primary and secondary health facilities to and expand its cadre of CHWs (accompagnateurs) to support adherence to therapy and retention in care. Furthermore, PIH used the attention and funding for HIV to improve the staffing, supply chain and community outreach (through paid CHWs) needed to improve primary health care and increase the utilization of basic health services. This stronger primary care platform was the ideal setting to launch provider initiated HIV testing which achieved a high level of population-based coverage of HIV services [8, 31]. PIH trained and paid CHWs, or *accompagnateurs*, to overcome structural barriers to ART adherence [32]. Each patient on ART was paired with an *accompagnateur*, who made home visits to observe ingestion of medication and helped address social and economic needs, from housing to education. Paid, full-time CHWs are crucial in such rural settings, and often spend 5 h per day to reach patients in their homes. The PIH community-based health care delivery model, informed by a biosocial perspective of health, also addresses social drivers of disease by offering food support and conditional cash transfers for transportation to clinics [3, 33].

**Fig. 1:**
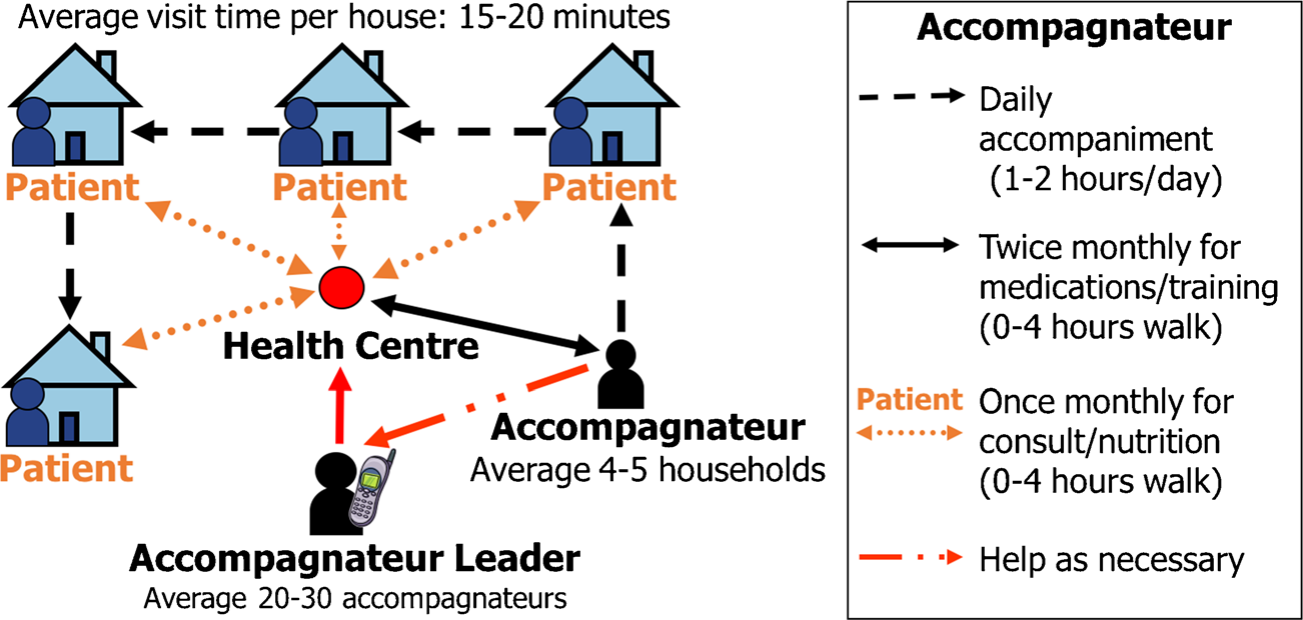
Partners In Health model of accompaniment

**Fig. 2:**
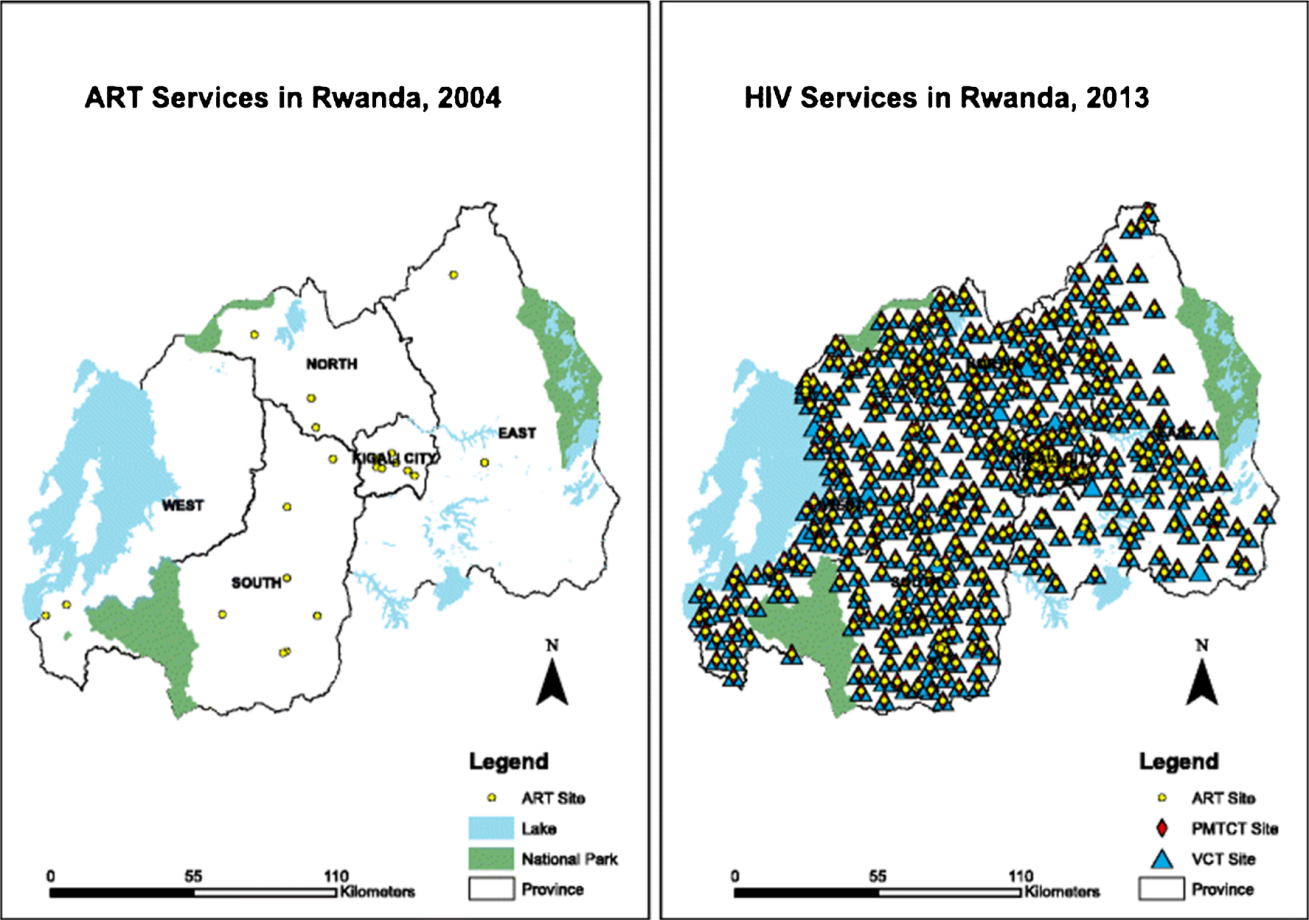
Decentralization of HIV services in Rwanda in 2004 (*left*) and 2013 (*right*). Used with permission from Dr. Nsanzimana at the Rwanda Biomedical Centre (RBC). *Source*: Nsanzimana S, Prabhu K, McDermott H, Karita E, Forrest JI, Drobac P, Farmer P, Mills EJ, Binagwaho A. Improving health outcomes through concurrent HIV program scale-up and health system development in Rwanda: 20 years of experience. BMC Med. 2015 Sep 9;13:216. Data from Institute of HIV/AIDS Disease Prevention & Control, Rwanda Biomedical Centre

It was largely due to the success of PIH’s work in Haiti that PIH decided, in 2005, to support the government of Rwanda (and later those of Lesotho and Malawi) in expanding access to ART services to very rural populations. In each of these countries, HIV monies were leveraged to support health systems that could delivery primary health care as well as HIV testing and ART. In each country, care is supported by a network of CHWs specifically focused on the accompaniment of patients with HIV and tuberculosis (Fig. 1). This approach was critical in informing the Rwandan rural health strategy. In 2015, investigators from the government of Rwanda, PIH, and other groups reviewed the history of community-based ART scale-up in Rwanda and showed how the national HIV response yielded benefits to the country’s overall health system; Fig. 2 illustrates the successful scale-up of ART services, which now reach more than 150,000 patients in Rwanda [34]. To achieve such successes, Rwandan health authorities have relied heavily on task shifting: nurses run primary health care clinics and prescribe HIV treatment. The government has also invested significantly in a large and well-trained community health workerforce, incentivizing more than 45,000 CHWs and engaging them in the delivery of ART and the performance of other health systems activities such as integrated management of childhood illness. These providers have been found to be highly engaged at the community level, though challenges such as high workload and inadequate supervision have also been seen [35].

In many parts of the world, CHWs bear a large burden of health programming, from promotion of hygiene and sanitation to treatment of diarrheal diseases and malaria; supervision of the breadth of activities with which CHWs are tasked is often poor. Because tuberculosis and HIV represent a significant disease burden in PIH catchment areas and supervision is focused on adherence to treatment and follow-up, PIH *accompagnateurs* in Rwanda, Haiti, Lesotho, and Malawi were initially recruited to focus specifically on HIV and tuberculosis care delivery. This focus enabled CHWs to be more effective in addressing the local burden of disease, rather than diffusing their efforts across a breadth of prevention activities. In Rwanda, PIH has documented that contact with CHWs supports treatment adherence and retention in HIV care and improves patients’ quality of life [36, 37]. The association between community-based accompaniment and psychosocial health was recently studied by Thompson et al. in a prospective cohort study of 610 HIV-infected adults initiated on ART. Both cohorts were initiated on ART, with one cohort additionally receiving community-based accompaniment: nutritional and socioeconomic supplements and daily home visits by an *accompagnateur* who provided social support and observed ingestion of ART. A multivariate analysis found that the addition of community-based accompaniment to standard HIV care was associated with an additional 44 % reduction in depression prevalence and an improvement in perceived mental and physical health quality of life [38]. Another prospective study, performed by PIH in rural Rwanda, followed 578 HIV-infected adults initiating ART in 2007 to 2008 with or without community-based accompaniment; besides patients with low CD4 cell counts, those receiving such accompaniment had significantly lower odds of poor clinical outcomes such as unsuppressed viral load, loss to follow-up, treatment default, or death within the first year of ART initiation [39].

## Community-Based ART as a Platform for Care Delivery Toward Universal Health Coverage

The PIH experience with community-based ART has gone far beyond simply addressing the HIV burden. Decentralization of HIV care has relied on well-trained professionals, a steady drug supply, laboratory services, and information systems. CHWs involved in HIV care have become experts in active case finding of vulnerable patients and in the long-term care of those with chronic diseases. To this end, PIH has leveraged the platform of community-based ART, emphasizing decentralization and CHWs, to meet the much broader spectrum of disease afflicting the poor. In Haiti, it has demonstrated the importance of applying HIV data systems to improve the quality of routine health services [40]. Specifically, investigators found that a national HIV quality improvement program, known as HIVQUAL, used to expand quality improvement capacity at 10 PIH-supported community primary health centers and two PIH-supported district hospitals significantly improved CD4 monitoring, tuberculosis screening, HIV treatment, and the prevention of mother-to-child transmission. They also documented the spread of quality improvement initiatives to other areas of inpatient and outpatient care, including tuberculosis, maternal health, and inpatient services in all 12 PIH-supported facilities. Also in Haiti, the community-based delivery platform developed through PIH’s experience in HIV care has served as the foundation for the provision of mental health screening and services to youth [41].

Similarly, in rural Malawi, PIH colleagues designed, implemented, and evaluated a community-based program to treat 114 adult patients with HIV-associated Kaposi’s sarcoma, the most common cancer in the country. Patients were identified and referred for treatment in the rural district hospital and clinics; they were also accompanied by paid CHWs for adherence to and retention in ART and chemotherapy and for provision of psychosocial support. Median follow-up was 86 weeks; 88 patients remained alive and in care (77 %), 19 patients died (17 %), and just six patients were lost to follow-up (5 %). This highly successful program was possible, the authors note, because of the integration of cancer care within an existing and effective community-based ART program [42]. From the provision of care for chronic conditions diseases to the identification, treatment, and follow-up of patients with cancer [43–45], PIH’s community-based, health center-enriched, and hospital-linked delivery systems have enabled an effective response to both the burden of disease afflicting the poor and the gaps encountered in their care, while serving as platforms for training thousands of professionals and generating new knowledge to alleviate suffering caused by disease.

In 2008, as part of the World Health Organization’s “Maximizing Positive Synergies between Health Systems and Global Health Initiatives” project [46], PIH tested the assumption that utilizing HIV funds to both scale up community-based ART and strengthen primary health care could at once deliver improved HIV care outcomes and increase primary care utilization. This work of health systems strengthening has culminated in the development of the PIH Universal Health Coverage (PIH-UHC) matrix, which maps universal health coverage targets based on the local burden of disease. Between 2014 and 2015, the PIH-UHC matrix has been used to support the Ministry of Health of Lesotho in its efforts to reform the health care sector toward universal health coverage. Since October 2014, 70 primary care clinics in Lesotho have been redesigned based on the PIH-UHC matrix. A preliminary analysis of data from these 70 facilities shows a more than threefold increase in utilization of services in the outpatient, antenatal, HIV, and tuberculosis clinics and facility-based delivery. Such work has significant implications for the development of health systems in post-Ebola West Africa and for financing efforts toward universal health coverage [47].

## Conclusion

Community-based ART programs have achieved remarkable results in expanding access to ART in resource-poor settings. They have also promoted retention in care and catalyzed efforts to build health systems that respond effectively to the complete burden of disease. However, much remains to be done. In 2014, the Joint United Nations Program on HIV/AIDS (UNAIDS) launched a Fast-Track strategy to accelerate the global AIDS response and called for the achievement of the 90-90-90 targets by 2020: that 90 % of people living with HIV will know their HIV status; that 90 % of those diagnosed will received sustained ART; and that 90 % of those on ART will have viral suppression [48]. UNAIDS argues that these targets, if achieved, would help end the global AIDS epidemic by 2030 through reduced transmission from HIV-infected persons. Furthermore, the Sustainable Development Goals, adopted by the United Nations in September 2015, include a call for Universal Health Coverage (UHC). The World Health Organization states, “Universal Health Coverage exists when all people receive the quality health services they need without suffering financial hardship” [49]. The UNAIDS Fast-Track strategy and the UHC agenda are mutually reinforcing, and their achievement will depend substantially on robust systems of community-based care. The global scale-up of ART and related HIV services offers a valuable set of lessons to achieve these aspirational goals.
